# Overall survival and subsequent therapy patterns in Japanese patients with ER+/HER2− advanced breast cancer treated with palbociclib plus letrozole in the first-line setting: a final analysis

**DOI:** 10.1007/s12282-025-01760-0

**Published:** 2025-08-21

**Authors:** Masato Takahashi, Hiroyuki Yasojima, Tomofumi Osako, Kenichi Inoue, Masahiro Kawashima, Hideki Maeda, Mitsuya Ito, Yasuaki Sagara, Kan Yonemori, Masaya Hattori, Naohito Yamamoto, Yasuaki Muramatsu, Akiko Matsui, Norikazu Masuda

**Affiliations:** 1 Breast Surgery, Hokkaido University Hospital, Sapporo, Hokkaido Japan; 2 Department of Surgery, Breast Oncology, NHO Osaka National Hospital, Osaka, Japan; 3 Breast Center, Kumamoto Shinto General Hospital, Kumamoto, Japan; 4 Department of Breast Care, Saitama Medical Center, Saitama, Japan; 5 Department of Breast Surgery, Graduate School of Medicine, Kyoto University, Kyoto, Japan; 6 Department of Breast Surgery, National Hospital Organization Hokkaido Cancer Center, Sapporo, Hokkaido Japan; 7 Breast Surgery, Hiroshima City Hiroshima Citizens Hospital, Hiroshima, Japan; 8 Breast Surgery, Sagara Hospital, Kagoshima, Japan; 9 Medical Oncology, National Cancer Center Hospital, Tokyo, Japan; 10 Aichi Breast Clinic Hirabari, Nagoya, Japan; 11 Department of Breast Oncology, Aichi Cancer Center, Nagoya, Japan; 12 Division of Breast Surgery, Chiba Cancer Center, Chiba, Japan; 13 Oncology Medical Affairs , Pfizer Japan Inc, Tokyo, Japan

**Keywords:** Palbociclib, Breast cancer, Japan, Overall survival, Subsequent therapies

## Abstract

**Background:**

An open-label, single-arm, multicenter Japanese phase 2 study (J-Ph2) found first-line palbociclib plus letrozole to be effective and tolerable in postmenopausal Japanese women with estrogen receptor-positive/human epidermal growth factor receptor 2-negative (ER+/HER2–) advanced breast cancer (ABC), but overall survival (OS) data were immature. Here, we report the final analysis of a follow-up study of J-Ph2 evaluating OS and subsequent therapy.

**Methods:**

Patients (*N* = 42) who participated in J-Ph2 were included in this follow-up study. Primary endpoint was OS; other endpoints included chemotherapy-free survival (CFS) and type and duration of subsequent therapy. Median OS, CFS, and duration of subsequent therapy were estimated using the Kaplan–Meier method; outcomes were stratified by baseline demographic, disease characteristics, and type of second-line therapies.

**Results:**

At median follow up of 101.0 months, median OS was 85.4 months (95% CI, 64.3–not estimable) and median CFS was 69.1 months (95% CI, 24.2–85.4). Eighty-one percent of patients (34/42) received a second-line therapy; of those, 85.3% (29/34) received endocrine-based therapy and 8.8% (3/34) received chemotherapy. Median duration of second-line therapy was 7.6 months. Sixty-nine percent of patients (29/42) received a third-line therapy; of those, 58.6% (17/29) received endocrine-based therapy and 31.0% (9/29) received chemotherapy; median duration of third-line therapy was 6.0 months.

**Conclusion:**

This analysis showed a median OS of  > 7 years with first-line palbociclib plus letrozole in Japanese patients with ER+/HER2– ABC. Patient demographics, disease characteristics, and subsequent therapy decisions may have contributed to the extended median OS observed in this study.

**Clinical trial registration:**

ClinicalTrials.gov, NCT04735367.

**Supplementary Information:**

The online version contains supplementary material available at 10.1007/s12282-025-01760-0.

## Introduction

Breast cancer is common and increasing among Japanese women [1, 2]. The recommended first-line treatment for postmenopausal women with hormone receptor-positive/human epidermal growth factor receptor 2-negative (HR+/HER2−) advanced breast cancer (ABC) in Japan is a cyclin-dependent kinase 4/6 inhibitor (CDK4/6i) plus an aromatase inhibitor [3].

Palbociclib is a first-in-class CDK4/6i that prolonged progression-free survival (PFS) in clinical trials enrolling largely Western patient populations [4–7]. Similarly, in the PALOMA-2 study, palbociclib plus letrozole significantly prolonged PFS compared with placebo plus letrozole (27.6 vs 14.5 months; hazard ratio [HR], 0.563, *P* < 0.0001) [5]; however, statistically significant prolongation of overall survival (OS) was not observed (53.9 vs 51.2 months; HR, 0.96) [7]. Subgroup analysis showed a better trend for OS in the Asia Pacific region (73.4 vs 55.1 months; HR 0.74; 95% CI 0.41–1.36) [7].

Palbociclib was approved in Japan for treatment of unresectable or recurrent breast cancer in 2017 [8]. An open-label, single-arm, multicenter, Japanese phase 2 study (J-Ph2; NCT01684215) investigated the efficacy and safety of first-line palbociclib plus letrozole in postmenopausal Japanese women with estrogen receptor-positive (ER+)/HER2− ABC, reporting a median PFS of 35.7 months (95% CI 21.7–46.7) [9]. However, OS data were immature at the end of the study. An extended follow-up study of J-Ph2 reported, at an interim analysis, a median OS of 85.4 months (95% CI 64.3– not estimable [NE]) after a median follow-up time of 89.7 months [10]. Here, we report the final analysis of OS and use of subsequent therapies in this extended follow-up J-Ph2 study.

## Patients and methods

### Study design and patients

This retrospective, multicenter, open-label, observational study (NCT04735367) was a planned follow up to J-Ph2 designed to evaluate OS and describe the type and duration of subsequent therapies in postmenopausal Japanese women with ER+/HER2− ABC treated with first-line palbociclib plus letrozole [9–11]. Thirteen institutions participated in the study [12]. A complete list of inclusion and exclusion criteria have been published [11]. This follow-up study was approved by each participating center’s Institutional Review Board and was conducted according to applicable local laws and regulatory requirements, the Ethical Guidelines for Medical and Health Research Involving Human Subjects issued by the Minister of Health, and Labour and Welfare. Informed written consent for continued participation in this study was obtained for all living participants; for participants who had died prior to this study, participants’ legal representatives were notified and given the opportunity to refuse data collection.

Patients received oral palbociclib 125 mg per day on a 21-days on/7-days off cycle with oral letrozole 2.5 mg per day continuously. Treatment was originally managed by clinicians according to the J-Ph2 protocol [11] and subsequently according to the Japanese palbociclib label guidelines. The primary endpoint was OS, defined as the time from first dose of study treatment in J-Ph2 to date of death due to any cause. Other endpoints included chemotherapy-free survival (CFS), defined as the time from first dose of study treatment in J-Ph2 to start of first subsequent chemotherapy or death due to any cause, and the type and duration of subsequent therapy.

### Statistical analyses

The statistical analysis set included all treated patients of J-Ph2; final analysis was based on the data cutoff date of 29 September 2023. Patient demographics and disease characteristics were summarized with descriptive statistics. Median OS, CFS, and duration of subsequent therapy were summarized using Kaplan−Meier methods, and median event time and 95% CIs were estimated. Outcomes were also assessed by subgroups, including metastatic status at disease progression (non-visceral vs visceral [patients with liver, lung, or pleural metastases] disease), treatment-free interval (TFI) from completion of adjuvant therapy (de novo disease, TFI ≤ 12 months, or TFI > 12 months), presence of new lesions at disease progression, duration of first-line palbociclib plus letrozole use (< 24 months vs ≥ 24 months), and second-line therapies.

## Results

### Patient population

A total of 42 patients were treated in J-Ph2. At baseline, patients had a median age of 62.5 years; 92.9% of patients had an ECOG PS of 0; 47.6% had visceral metastases; and 19.0% had TFI ≤ 12 months (similar to patients in the palbociclib arm of PALOMA-2 [7]: patients had a median age of 62.0 years; 57.9% of patients had an ECOG PS of 0, 48.2% had visceral metastases, and 22.1% had TFI ≤ 12 months), 47.6% had a TFI > 12 months, and 33.3% had de novo disease (Supplementary Table S1).

### Overall survival and chemotherapy-free survival

**Fig. 1:**
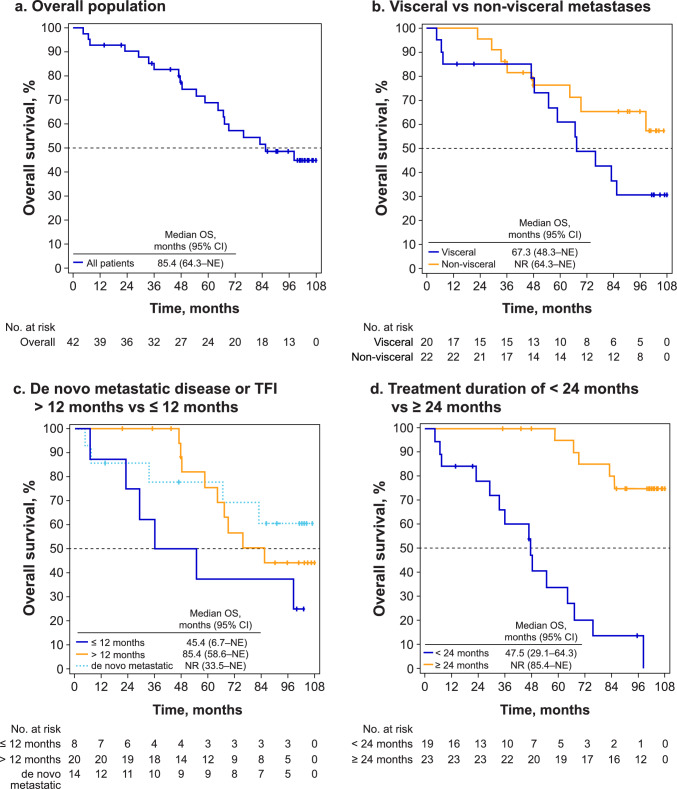
Kaplan−Meier plot of overall survival in the overall population (**a**) and by type of metastases (**b**), treatment-free interval (**c**), and duration of first-line palbociclib treatment (**d**). *CDK4/6i* cyclin-dependent kinase 4/6 inhibitor, *CI* confidence interval, *ET* endocrine therapy, *NE* not estimable, *NR* not reached, *OS* overall survival, *TFI* treatment-free interval

At final analysis, with a median follow-up of 101.0 months, median OS remained at 85.4 months (95% CI 64.3–NE; Fig. 1a). For patients with visceral metastases, median OS was 67.3 months, while it was not reached (NR) in those with non-visceral metastases (Fig. 1b). For patients with TFI > 12 months, median OS was 85.4 months and was 45.4 months for those with TFI ≤ 12 months; median OS was NR for those with de novo metastatic disease (Fig. 1c). For patients with treatment duration of first-line palbociclib plus letrozole < 24 months median, OS was 47.5 months, while it was NR for those with treatment duration ≥ 24 months (Fig. 1d).

Median CFS remained at 69.1 months (95% CI 24.2–85.4; Supplementary Figure S1A). Subgroup analyses of CFS are provided in Supplementary Figures S1B and S1C.

### Subsequent therapies

**Fig. 2:**
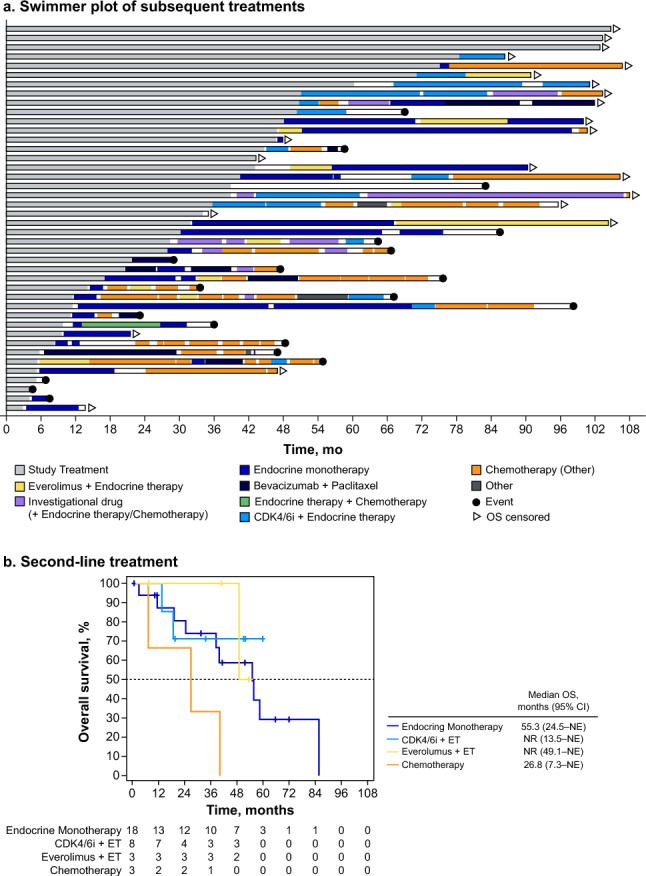
Swimmer plot of subsequent therapies in the overall population (**a**) and Kaplan−Meier curve of overall survival by second-line therapy (**b**) *CDK4/6i* cyclin-dependent kinase 4/6 inhibitor, *CI* confidence interval, *ET* endocrine therapy, *NE* not estimable, *NR* not reached, *OS* overall survival

At final analysis, 7.1% of patients (3/42) were still receiving palbociclib plus letrozole, 81.0% (34/42) moved to a subsequent therapy, and 11.9% (5/42) terminated study treatment without subsequent treatment (Fig. 2a). Of patients who received a second-line treatment, 85.3% (29/34) received endocrine-based therapy as their second-line treatment; 8.8% (3/34) received chemotherapy, and 5.9% (2/34) received other investigational therapies (Table 1). Median OS by type of second-line therapy is shown in Fig. 2b.

**Table 1:** Type of subsequent therapy overall and by subgroups

Subsequent therapy, *n* (%)	Overall (*N* = 42)	Status of metastases at disease progression^e^		New lesions at disease progression^e^		Duration of first-line treatment	
		Visceral (*n* = 16)	Non-visceral (*n* = 13)	Yes (*n* = 17)	No (*n* = 12)	< 24 months (*n* = 19)	≥ 24 months (*n* = 23)
**No subsequent therapy**	8	1	1	2	0	2	6
Study treatment ongoing	3 (37.5)	0	0	0	0	0	3 (50.0)
Study treatment terminated	5 (62.5)	1 (100)	1 (100)	2 (100)	0	2 (100)	3 (50.0)
**Second-line therapy**^a^	34	15	12	15	12	17	17
Endocrine-based therapy	29 (85.3)	12 (80.0)	10 (83.3)	12 (80.0)	10 (83.3)	13 (76.5)	16 (94.1)
ET alone	18 (52.9)	8 (53.3)	5 (41.7)	7 (46.7)	6 (50.0)	11 (64.7)	7 (41.2)
CDK4/6i + ET^b^	8 (23.5)	3 (20.0)	3 (25.0)	4 (26.7)	2 (16.7)	1 (5.9)	7 (41.2)
Everolimus + ET	3 (8.8)	1 (6.7)	2 (16.7)	1 (6.7)	2 (16.7)	1 (5.9)	2 (11.8)
Chemotherapy^c^	3 (8.8)	2 (13.3)	1 (8.3)	3 (20.0)	0	3 (17.6)	0
Other^c^	2 (5.9)	1 (6.7)	1 (8.3)	0	2 (16.7)	1 (5.9)	1 (5.9)
**Third-line therapy**^a^	29	13	9	11	11	14	15
Endocrine-based therapy	17 (58.6)	6 (46.2)	6 (66.7)	7 (63.6)	5 (45.5)	6 (42.9)	11 (73.3)
ET alone	9 (31.0)	4 (30.8)	2 (22.2)	3 (27.3)	3 (27.3)	5 (35.7)	4 (26.7)
CDK4/6i + ET	5 (17.2)	1 (7.7)	2 (22.2)	2 (18.2)	1 (9.1)	1 (7.1)	4 (26.7)
Everolimus + ET	3 (10.3)	1 (7.7)	2 (22.2)	2 (18.2)	1 (9.1)	0	3 (20.0)
Chemotherapy	9 (31.0)	6 (46.2)	2 (22.2)	4 (36.4)	4 (36.4)	6 (42.9)	3 (20.0)
Other^d^	3 (10.3)	1 (7.7)	1 (11.1)	0	2 (18.2)	2 (14.3)	1 (6.7)

^a^Percentages calculated based on total number receiving second-/third-line therapy

^b^4 patients received abemaciclib, and 4 patients continued to receive palbociclib

^c^All patients received bevacizumab + paclitaxel

^d^Investigational drug + ET/chemotherapy or ET + chemotherapy

^e^13 patients were excluded because they discontinued treatment or were censored for reasons other than disease progression (e.g., adverse events, lost to follow-up)

*CDK4/6i* cyclin-dependent kinase 4/6 inhibitor, *ET* endocrine therapy

**Fig. 3:**
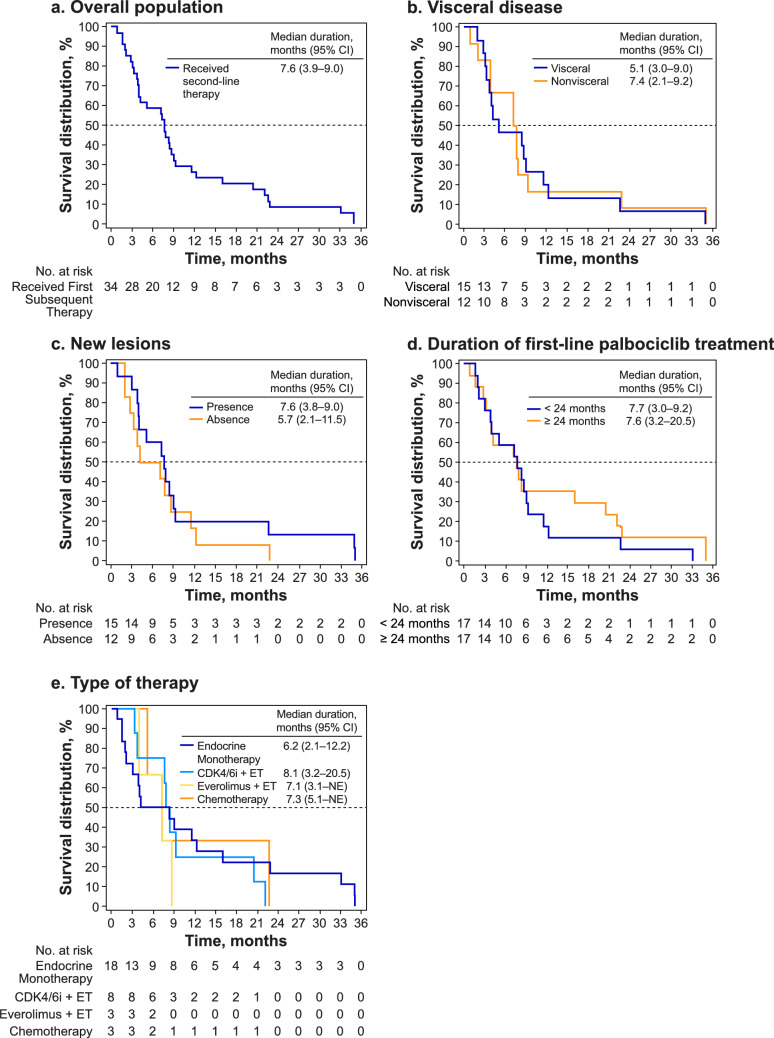
Kaplan−Meier plot of duration of second-line therapy overall (**a**), by visceral status at disease progression (**b**), new lesions at disease progression (**c**), duration of first-line palbociclib treatment (**d**), and type of second-line therapy (**e**). *CDK4/6i* cyclin-dependent kinase 4/6 inhibitor, *CI* confidence interval, *ET* endocrine therapy, *NE* not estimable, *OS* overall survival

Median duration of second-line therapy in the overall cohort was 7.6 months (95% CI 3.9–9.0; Fig. 3a). Median duration in patients with visceral metastases at disease progression was 5.1 months and 7.4 months in patients with non-visceral metastases at disease progression (Fig. 3b). In patients with new lesions at disease progression, median duration was 7.6 months and 5.7 months in patients with no new lesions at disease progression (Fig. 3c). Patients with duration of first-line palbociclib treatment of < 24 months had a median duration of second-line therapy of 7.7 months; for patients with duration of first-line palbociclib treatment ≥ 24 months, median duration was 7.6 months (Fig. 3d). When analyzed by type of second-line therapy (Fig. 3e), median durations of treatment were 8.1 months (95% CI 3.2–20.5) for CDK4/6i + endocrine therapy (ET), 7.3 months (95% CI 5.1 − NE) for chemotherapy, 7.1 months (95% CI 3.9 − NE) for everolimus + ET and 6.2 months (95% CI 2.1–12.2) for ET alone.

Among those patients who received a third-line therapy (69.0%; 29/42), 58.6% (17/29) received an endocrine-based therapy (without chemotherapy), 31.0% (9/29) received chemotherapy alone, and 10.3% (3/29) received other investigational therapy (Table 1). Median duration of the third-line therapy was 6.0 months (95% CI 3.3–14.9). The proportion of patients using different types of subsequent therapies were similar when stratified by presence of visceral metastases at disease progression or presence of new lesions at disease progression, while a higher proportion of patients had endocrine-based subsequent therapy when duration of first-line palbociclib treatment was ≥ 24 months compared with < 24 months (Table 1 and Supplementary Figure S2.

## Discussion

In this final analysis of the J-Ph2 extended follow-up study, Japanese patients with ER+/HER2− ABC who received first-line palbociclib plus letrozole had a median OS of 85.4 months and median CFS of 69.1 months after a median follow-up of 101.0 months. Although they cannot be directly compared, a strength of this open-label, Japanese study is that the median OS (85.4 months) with first-line palbociclib is longer than seen in randomized controlled trials primarily enrolling Western patients (PALOMA-2: 53.9 months [7]), and a United States-based, real-world study (P-REALITY X: 49.1 months) [13]. Similar to our J-Ph2 results, a Japanese, multicenter, real-world study of medical records from patients with HR+/HER2− ABC receiving first-line palbociclib plus ET reported that median OS was not reached after a median follow-up of 36.3 months [14]; median OS in patients with TFI > 12 months and de novo metastasis was not reached, whereas for patients with TFI ≤ 12 months, it was 50.1 months.

To explore why this study achieved such a long OS, we focused further investigation on subsequent treatments after first-line palbociclib. At the final analysis, 3 patients (7.1%) remained on study treatment, 81.0% of patients had received second-line treatment, and 69.0% had received third-line treatment. Most patients received hormone-based therapy as their second-line (85.3%) or third-line (58.6%) treatment. The proportion of patients receiving chemotherapy slightly increased by the time they transitioned to their third-line treatment. A greater proportion of patients received second-line endocrine-based therapy if they had received first-line palbociclib for ≥ 24 months (94.1%) compared with those with first-line duration < 24 months (76.5%).

The duration of second-line treatment was analyzed by subgroups based on factors considered when selecting subsequent treatment, including presence of new disease or visceral metastases at disease progression and duration of previous treatment. Notably, regardless of poor prognoses, there were no significant differences in second-line treatment duration for patients based on time to disease progression (short or long) or visceral metastatic status (with or without). This may be due to selecting the appropriate subsequent treatment for each patient (e.g., adding molecular-targeted drugs or shifting to chemotherapy for these populations) and careful adverse event management tailored to the patient’s condition, which may extend the duration of each line of treatment. However, all patients with duration of first-line treatment ≥ 24 months were on hormone based therapy as their second-line treatment, suggesting that this population may benefit from endocrine-based therapy past the first-line setting; this could have contributed to the longer median OS. Additionally, the treatment landscape after progression on CDK4/6i has shifted in recent years, including the recent approval in Japan of capivasertib for use in patients with HR+/HER2– MBC with alterations in *PIK3CA, AKT1, or PTEN* after progression on prior endocrine therapy [15]. Additionally, if trastuzumab deruxtecan is approved in the future in Japan for ultra-low HER2 + breast cancer [16], the landscape for post-CDK4/6i treatment will shift further. These patient management strategies and newly available targeted treatments may also have contributed to the longer OS of > 7 years.

However, all patients with duration of first-line treatment ≥ 24 months were on hormone-based therapy as their second-line treatment, suggesting that this population may benefit from endocrine-based therapy past the first-line setting; this could have contributed to the longer median OS. Previous studies show that Japanese patients have favorable prognoses with endocrine-based therapy; in a phase 3, Japanese clinical trial of exemestane compared with anastrozole, median OS for the anastrozole group was 60.1 months and was not reached in the exemestane group, with no OS differences observed in Kaplan–Meier plots [17].

Although patient backgrounds were not consistent across the different second-line treatment groups, and the small number of patients makes strict evaluation difficult, treatment duration for endocrine monotherapy in the second line was 6.2 months, while CDK4/6i and everolimus in combination with ET extended the treatment duration by approximately 1 to 2 months. Real-world evidence from a Japanese, multicenter, observational study (December 2017 − December 2020) also found most common therapies after first- (*n* = 426) and second-line (*n* = 267) palbociclib were endocrine-based therapies, with 60.7% and 61.1% of patients, respectively, having received them; 23.1% and 15.9% of patients, respectively, received ET with a CDK4/6 inhibitor [18]. Now that first-line treatment with CDK4/6i is the standard of care, it is important to further investigate which new treatment options can extend OS and the importance of selecting treatments based on biomarkers in such cases. It has also been reported that second-line therapy after first-line treatment with CD4/6i shows better prognosis when combined with molecular targeted drugs compared to ET [19, 20].

### Limitations

This was a single-arm study with no control group, limiting the interpretation of the results, particularly for time-to-event endpoints. The small sample size may make it difficult to draw definitive conclusions as it may not be representative of the larger Japanese population. Known genetic poor prognostic factors of tumors (e.g., mutations in *ESR1*, *PIK3CA*, *AKT1*, and *PTEN*), which are now standard to evaluate in patients with ABC, were not confirmed in this study. In addition, Japan-specific factors not measured in this study could have influenced the prolonged overall survival observed. Finally, as portions of this study were retrospective and observational, there is a potential for missing, inaccurate, or incomplete data.

## Conclusions

In this final analysis of the J-Ph2 follow-up study, Japanese women with ER+/HER2– ABC treated with first-line palbociclib plus letrozole had a median OS of > 7 years. The increase in overall OS compared to previous studies may be due to proper selection of subsequent therapies after palbociclib treatment, adverse event management, and an increase in treatment duration. This analysis also provides insight into real-world subsequent treatment patterns following palbociclib plus letrozole therapy in HR+/HER2– ABC, adding to the growing body of evidence [21] supporting the use of palbociclib as a first-line treatment of ER+/HER2– ABC in Japanese women.

## Supplementary Information

Below is the link to the electronic supplementary material.

Supplementary file1 (DOCX 177 KB)

## Data Availability

Upon request, and subject to certain criteria, conditions, and exceptions (see https://www.pfizer.com/science/clinical-trials/trial-data-and-results for more information), Pfizer will provide access to individual de-identified participant data from Pfizer-sponsored global interventional clinical studies conducted for medicines, vaccines, and medical devices (1) for indications that have been approved in the US and/or EU or (2) in programs that have been terminated (i.e., development for all indications has been discontinued). Pfizer will also consider requests for the protocol, data dictionary, and statistical analysis plan. Data may be requested from Pfizer trials 24 months after study completion. The de-identified participant data will be made available to researchers whose proposals meet the research criteria and other conditions, and for which an exception does not apply, via a secure portal. To gain access, data requestors must enter into a data access agreement with Pfizer.
