# Supplementary material for: Overall survival and subsequent therapy patterns in Japanese patients with ER+/HER2− advanced breast cancer treated with palbociclib plus letrozole in the first-line setting: a final analysis
Source: Breast Cancer. 2025 Aug 21;32(6):1482–90. doi: 10.1007/s12282-025-01760-0 (PMC12552339; doi:10.1007/s12282-025-01760-0)
Supplement: Supplementary file 1 — Supplementary file1 (DOCX 177 KB) [file 12282_2025_1760_MOESM1_ESM.docx]

# **Supplementary Material**

**Table S1** Patient demographics and disease characteristics

| **Demographic or disease characteristic** | **Population**  **(N = 42)** |
| --- | --- |
| Age, median (range), years | 62.5 (43–84) |
| Weight, median (range), kg | 50.4 (38.6–74.5) |
| ECOG PS, n (%)  0  1 | 39 (92.9)  3 (7.1) |
| Disease site, n (%) |  |
| Visceral | 20 (47.6) |
| Non-visceral | 22 (52.4) |
| Bone only | 6 (14.3) |
| Treatment-free interval, n (%)  ≤ 12 months  > 12 months  de novo metastatic | 8 (19.0)  20 (47.6)  14 (33.3) |
| Prior (neo)adjuvant therapies, n (%)  Hormone therapy  Chemotherapy | 27 (64.3)  20 (47.6) |
| Ki67-positive expression, n (%)  ≤ 20%  > 20% | 19 (45.2)  23 (54.8) |

*ECOG PS* Eastern Cooperative Oncology Group performance status

**Figure S1** Kaplan-Meier plot of CFS in overall population (a), by visceral metastases (b), and de novo metastatic disease or treatment-free interval (c).


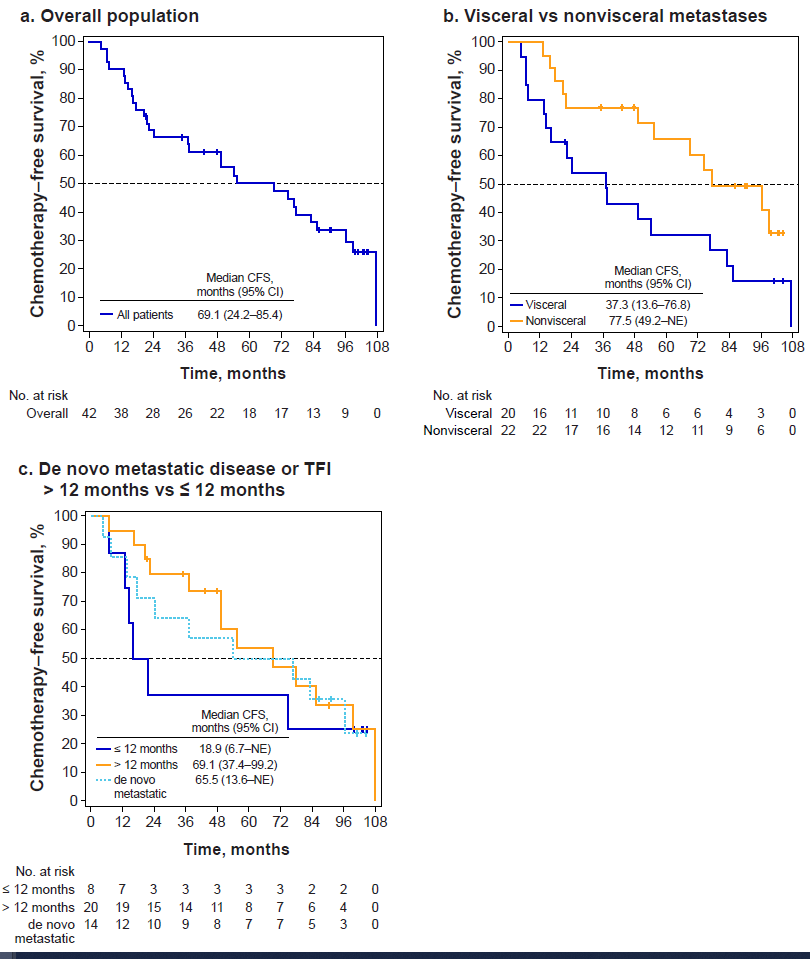


*CFS* chemotherapy-free survival, *CI* confidence interval, *NE* not estimable, *TFI* treatment-free interval

**Figure S2** Swimmer plot of subsequent therapy by status of metastases at disease progression (a), new lesions at disease progression (b), and duration of first-line palbociclib treatment (c).


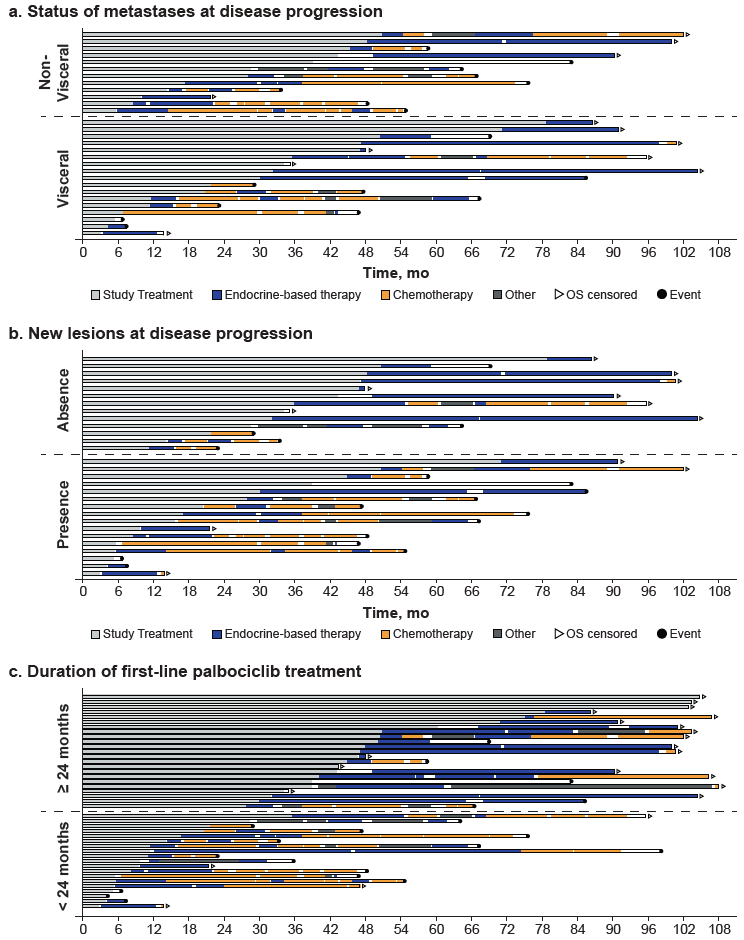


*OS* overall survival
